# Epidemiology of hip fracture in Qatar and development of a country specific FRAX model

**DOI:** 10.1007/s11657-022-01083-z

**Published:** 2022-03-18

**Authors:** Nabeel Abdulla, Omar Suhail Alsaed, Abdo Lutf, Fiaz Alam, Ibrahim Abdulmomen, Samar Al Emadi, Nicholas C. Harvey, Enwu Liu, Liesbeth Vandenput, Mattias Lorentzon, Eugene McCloskey, John A. Kanis, Helena Johansson

**Affiliations:** 1 Division of Rheumatology, Department of Medicine, Hamad Medical Corporation, Alrayyan Street, PO BOX 3050, Doha, Qatar; 2 MRC Lifecourse Epidemiology Unit, University of Southampton, Southampton, UK; 3 NIHR Southampton Biomedical Research Centre, University of Southampton and University Hospital Southampton NHS Foundation Trust, Southampton, UK; 4 Mary McKillop Institute for Health Research, Australian Catholic University, Melbourne, Australia; 5 Centre for Bone and Arthritis Research, Department of Internal Medicine and Clinical Nutrition, Institute of Medicine, Sahlgrenska Academy, University of Gothenburg, Gothenburg, Sweden; 6 Sahlgrenska Osteoporosis Centre, Institute of Medicine, University of Gothenburg, Gothenburg, Sweden; 7 Centre for Metabolic Bone Diseases, University of Sheffield, Sheffield, UK; 8 Department of Oncology and Metabolism, Mellanby Centre for Musculoskeletal Research, University of Sheffield, Sheffield, UK

**Keywords:** FRAX, Fracture probability, Epidemiology, Hip fracture, Qatar

## Abstract

***Summary*:**

Hip fracture data were retrieved from electronical medical records for the years 2017–2019 in the State of Qatar and used to create a FRAX® model to facilitate fracture risk assessment. Hip fracture rates were comparable with estimates from Saudi Arabia, Abu Dhabi, and Kuwait but fracture probabilities varied due to differences in mortality.

**Objective:**

This paper describes the epidemiology of osteoporotic fractures in the State of Qatar that was used to develop the country-specific fracture prediction FRAX® tool.

**Methods:**

Hip fracture data were retrieved from electronic medical records for the years 2017–2019 in the State of Qatar. The age and sex specific incidence of hip fracture in Qatari residents and national mortality rates were used to create a FRAX® model. Fracture probabilities were compared with those from neighboring countries having FRAX models.

**Results:**

Hip fracture rates were comparable with estimates from Saudi Arabia, Abu Dhabi and Kuwait. In contrast, probabilities of a major osteoporotic fracture or hip fracture were lower in Qatar than in Kuwait but higher than those in Abu Dhabi and Saudi Arabia due to differences in mortality.

**Conclusion:**

The FRAX model should enhance accuracy of determining fracture probability among the Qatari population and help guide decisions about treatment.

## Introduction

Osteoporosis is operationally defined on the basis of bone mineral density (BMD) assessment by dual-energy X-ray absorptiometry (DXA), with recent refinements of the description focusing on measurements at the femoral neck as a reference standard [1]. The WHO-defined T-score of − 2.5 or lower, originally designed for classification in epidemiological studies, has since been widely adopted as both a diagnostic and intervention threshold. A principal difficulty for fracture risk assessment is that, whereas this threshold has high specificity it has low sensitivity, such that the majority of fragility fractures occur in individuals with BMD values above the osteoporosis threshold [2]. A second problem is that the risk of fracture varies markedly worldwide [3], much more so than can be accounted for by variations in BMD. Thus, a given BMD has a markedly different significance in different countries in terms of fracture risk [4].

Many risk factors have been identified over the last two decades that contribute to fracture risk, at least partly if not wholly independently of DXA BMD. These include age, sex, a prior fracture, a family history of fracture, and lifestyle risk factors such as physical inactivity and smoking [5]. These and other factors have been combined in analyses of individual cohort studies to develop algorithms and scores to characterize future risk at the level of an individual. Such independent risk factors used with BMD can enhance fracture risk assessment; additionally, the incorporation of risk factors that correlate with BMD (e.g., age, fracture, body mass index (BMI)) can also facilitate fracture risk assessment in situations in which DXA is not available. These were the considerations underlying the development of the FRAX® tool, which was devised by the former WHO Collaborating Centre at the University of Sheffield [Kanis 2007, 2008b]. FRAX (https://www.sheffield.ac.uk/FRAX/), recommended in more than 100 national and international guidelines [6], computes the 10-year probability of low energy fractures based on several common clinical risk factors and, optionally a DXA scan result [5, 7]. Specifically, FRAX models compute the probabilities of major osteoporotic and hip fracture derived from the risk of fracture and the competing risk of death, both of which vary from country to country. The development of fracture risk assessment tools has enabled a step change in the management of osteoporosis as patients can now be selected for therapy on the basis of absolute fracture risk rather than BMD T-score alone.

The development of country-specific FRAX models requires information on fracture incidence and death. No FRAX model is available for Qatar due to the paucity of appropriate epidemiological data [8]. This report describes the acquisition of data for the creation of a country specific FRAX model for the State of Qatar.

## Methods

The State of Qatar is located in the Middle East, comprising a peninsula in the Arabian Gulf on the northeastern coast of the Arabian Peninsula. Its land border is with Saudi Arabia. In 2020, Qatar’s total population was 2.8 million, of which approximately 12% were Qatari citizens and the remainder expatriates [9].

Hamad Medical Corporation (HMC) is the principal public health care provider in the state of Qatar with several facilities across the country. Hip fracture data were extracted from HMC electronic medical records by using ICD 10 codes (S72.0, S72.1, S72.2) from January 2017 to December 2019. Although there are private hospitals in Qatar, they do not undertake surgery for hip fractures so that hip fracture cases presenting to HMC represent national data.

Fracture cases were recorded from the age of 40 years since this is the age from which FRAX is used to calculate fracture probabilities. Multiple admissions by the same patient for the same fracture were excluded to avoid duplication. For the present analysis, we included patients irrespective of the degree of trauma. The reason for their inclusion is that classification of high and low energy fractures is inconsistent and arbitrary. Additionally, high-trauma and non-trauma fractures show similar relationships with low BMD and future fracture risk [10, 11]. We included Qatari citizens and expatriates in order to compare hip fracture rates.

Population estimates for Qatari citizens and expatriates in 2017 were based on data for 2017 in 5-year intervals [Qatar Population and Employment Projections 2017–2042—a framework for National Planning]. Data for 2018 and 2019 were estimated from population projections from 2017.

Possible differences in incidence between the Qatari citizens and the expatriate population were explored using age-standardized annual rates based on the population distribution of Qatari nationals. Age- and sex-specific fracture incidence was compared to data available for Abu Dhabi, Kuwait and Saudi Arabia.

### FRAX model

The data on hip fracture in Qatari nationals were used to construct the FRAX model. For other major osteoporotic fractures (MOF; clinical spine, forearm, and humeral fractures), it was assumed that the age- and sex-specific ratios of these fractures to hip fracture risk found in Sweden were comparable to those in Qatar. This assumption has been used for many of the FRAX models with incomplete epidemiological information. Available information suggests that the age- and sex-specific pattern of fracture is very similar in the Western world, Australia, and Eastern Europe [12–15].

The development and validation of FRAX have been extensively described [5, 6]. The risk factors used were based on a systematic set of meta-analyses of population-based cohorts worldwide and validated in independent cohorts with over 1 million patient-years of follow-up. The construct of the FRAX model for Qatar retained the beta coefficients of the risk factors in the original FRAX model, together with the smoothed incidence rates of hip fracture and mortality rates for Qatari nationals. National mortality rates for Qatari nationals for years 2018–2019 used data from the Planning and Statistics Authority [16]. Ten-year fracture probabilities were compared to those of the neighboring countries where a FRAX model was available (Saudi Arabia, Kuwait and Abu Dhabi).

In order to compare Qatari hip fracture probabilities with those in other regions of the world, the remaining lifetime probability of hip fracture from the age of 50 years was calculated for men and women, as described by Kanis et al. [17]. In the present analysis, values for Qatar were compared with those for Abu Dhabi, Botswana, Bulgaria, Canada, China (Hong Kong), Denmark, Finland, France, Germany, Greece, Hungary, Iran, Kazakhstan, Kuwait, Moldova, Morocco, Netherlands, Poland, Portugal, Romania, Russia, Singapore, South Africa, Spain, Sweden, Tunisia, Turkey, UK, Ukraine, USA, and Uzbekistan [18].

## Results

A total of 492 hip fracture cases were identified over the 3-year interval. Of these, 151 (31%) arose in Qatari nationals. Hip fracture rates were generally higher in women than in men and increased with age. Hip fracture rates were marginally lower in the Qatari population than in the entire population. When the Qatari population was compared to the expatriate population, age-standardized annual rates were not significantly different in men (68; 95% CI = 53–86/100,000 vs. 82; 95% CI = 65–102/100,000, respectively). In women, however, age-standardized annual rates were significantly lower in the Qatari population (74; 95% CI = 59–91/100,000 vs. 107; 95% CI = 89–128/100,000, respectively; *p* = 0.0082). Thus, for the development of the FRAX model, the incidence of Qatari nationals was used for both men and women (Table 1).

**Table 1:** Incidence of hip fracture per 100,000 and 95% confidence interval in the whole population and in Qatari nationals

	Entire population		Qataris	
	Male	Female	Male	Female
40–44	5.3 (3.6–7.4)	3.2 (1.0–7.5)	9.1 (1.1–33.1)	12.0 (2.5–35.2)
45–49	7.9 (5.5–11.1)	1.9 (0.2–6.9)	5.0 (0.1–28.1)	0.0 (0.0–16.6)
50–54	8.6 (5.5–13.0)	14.3 (6.8–26.3)	29.9 (9.7–69.9)	20.3 (5.5–51.9)
55–59	12.7 (8.0–19.3)	30.4 (16.6–51.0)	22.0 (4.5–64.3)	42.1 (16.9–86.7)
60–64	35.9 (23.6–52.2)	54.4 (30.4–89.8)	39.7 (10.7–101.7)	50.0 (18.3–108.8)
65–69	111.2 (77.9–154.0)	169.2 (108.4–251.9)	114.5 (46.0–236.0)	177.5 (91.7–310.3)
70–74	284.7 (201.4–390.8)	210.2 (126.4–328.3)	296.2 (161.8–497.3)	176.4 (84.5–324.4)
75–79	466.1 (314.4–665.6)	564.6 (375.1–816.3)	446.3 (230.4–780.0)	512.3 (286.4–845.4)
80 +	834.1 (612.8–1109.2)	968.7 (723.3–1270.5)	675.0 (406.1–1054.3)	885.8 (583.5–1289.2)

**Fig. 1:**
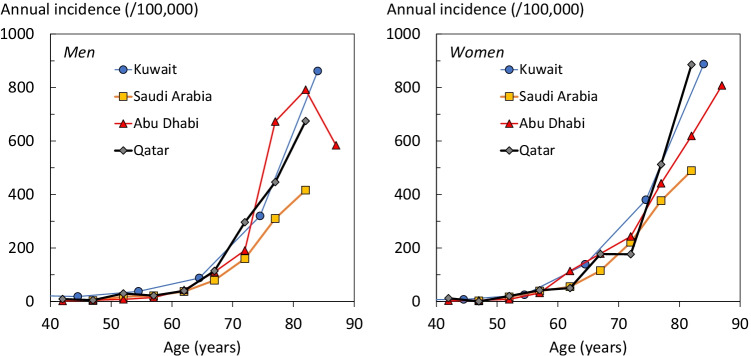
Annual incidence of hip fracture in men (left hand panel) and women (right panel) by age from Abu Dhabi, Kuwait, Qatar, and Saudi Arabia

Age-specific fracture rates were higher in women than in men except for the age intervals 45–54 and 70–74 years (Fig. 1) with a crude sex ratio (F/M) of 1.25. Hip fracture rates were similar to those reported in Kuwait, Saudi Arabia and Abu Dhabi.

**Fig. 2:**
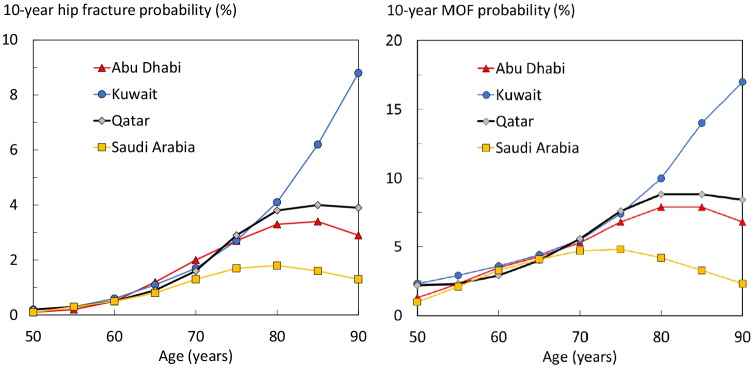
10-year probability of hip fracture (left hand panel) and major osteoporotic fracture (right panel) in women with no risk factors by age from Abu Dhabi, Kuwait, Qatar, and Saudi Arabia. Body mass index set to 25 kg/m^2^.

Probabilities of a hip fracture are shown for women in Fig. 2. Probabilities in the Qatari population rose with age up to the age of 80 years and plateaued thereafter due to the competing effect of mortality. A similar pattern of hip fracture probabilities was observed in Abu Dhabi. In contrast, hip fracture probabilities, similar at younger ages, rose progressively with age in Kuwait with no evidence of a plateau. In the case of Saudi Arabia, hip fracture probabilities, similar at younger ages, plateaued much earlier than in Qatar. Thus, there was a modest difference in hip fracture probability at the age of 50 years but a greater than sixfold range at age 90 years (8.8% in Kuwait and 1.3% in Saudi Arabia). The patterns for 10-year probabilities of a MOF were similar to those for hip fracture (see Fig. 2).

Lifetime probabilities for hip fracture are shown in Table 2. For Qatar, probabilities were approximately than 1 in 10 women, and similar to probabilities in Hungary and Bulgaria. As might be expected from the differences in mortality, probabilities were approximately double that estimated for Saudi Arabia.

**Table 2:** Life-time probability of hip fracture in the Qatari population at the age of 50 years compared with selected countries. Data from [18] unless otherwise indicated

Country	Life-time risk at 50 years (%)	
	Women	Men
Sweden	25.6	11.0
South Africa (white)	23.4	7.7
Denmark	23.0	11.3
France	19.3	5.9
China (Hong Kong)	17.7	7.6
USA (Caucasian)	16.1	7.5
Turkey	15.9	3.6
Canada	15.5	5.8
Greece	15.4	6.8
Uzbekistan	14.7	8.7
UK	14.4	5.0
Germany	14.2	5.3
Portugal	13.7	4.8
Finland	12.9	6.0
Kazakhstan	12.6	6.0
Spain	12.6	4.2
Netherlands	12.5	5.4
Singapore (Indian)	12.5	5.2
Bulgaria	11.2	4.4
Qatar^a^	11.0	8.8
Hungary	10.8	4.2
Poland	10.1	4.2
Moldova	9.3	5.7
Kuwait	9.2	7.6
Abu Dhabi	8.9	8.1
Iran	8.3	5.5
Russia	7.7	3.8
Romania	7.0	3.8
USA (black)	5.9	2.7
Ukraine	5.6	2.9
Saudi Arabia^b^	4.6	3.7
South Africa (Black)	4.5	1.9
Morocco	4.1	3.1
Botswana	1.1	1.4
Tunisia	0.7	0.7

^a^Present study

^b^JA Kanis, personal communication

## Discussion

This study documented the incidence of hip fractures in Qatar in order to permit the construction of a FRAX model. Qatari citizens were found to have a lower incidence of hip fracture than the expatriate population, and so the Qatari rates were used in the construct of the FRAX model. As expected, hip fractures rates were higher in women than in men (female/male ratio = 1.25). In both sexes, the incidence increased with age. In an international perspective, hip fracture incidence was low in both men and women [3]. It is of interest that the incidence of hip fracture was rather similar to that reported for Saudi Arabia, Kuwait, and Abu Dhabi. There was, however, a very marked difference in fracture probability between countries with advancing age. The explanation for the difference lies in the assumptions for mortality since fracture probability integrates the fracture hazard with the competing effect of mortality. Thus, the death hazard was highest with advancing age in Saudi Arabia, lowest in Kuwait and intermediate for Abu Dhabi and Qatar. These observations emphasize the importance of the death hazard as well as the fracture hazard in the determination of fracture probability.

A minority of countries that have a FRAX model also have robust information on the risk of other major osteoporotic fractures. In the absence of such information, FRAX models are based on the assumption that the age- and sex-specific pattern of these fractures is similar to that observed in Malmo, Sweden [13]. The assumption has been validated in studies from Canada [15], Iceland [14], USA [19], UK [20], Australia [10], and Eurasia [12] despite very marked differences in incidence [3]. This commonality of pattern is supported by register studies, which indicate that in those regions where hip fracture rates are high, so too is the risk of forearm fracture and spine fractures (requiring hospital admission) [21, 22]. Studies of incidence rather than prevalence of vertebral fracture confirm a much higher incidence of vertebral fracture in US blacks than whites [23].

Whereas the Qatari FRAX model permits the assessment of fracture probability in Qatari citizens, the question arises of how to assess fracture risk in expatriates. This community is ethnically very diverse and current evidence indicates that expatriates retain the risk characteristics of their country of origin [24, 25], so should be assessed as such.

The limitations of the present study relate predominately to the accuracy of the FRAX model. This in turn is dependent on the accuracy of the fracture and death hazards used in the construction of the FRAX model. Whereas death rates for the general population are likely to be robust, the number of hip fractures were few (*n* = 151) despite the national catchment and 3-year study interval. Thus, age- and sex-specific hip fracture rates are bound by wide confidence intervals. The quality of studies on hip fracture incidence are usually determined on the basis of national representation and duration of observation [3] and perhaps sample size should be added to these criteria. Thus, age- and sex-specific hip fracture rates were bound by wide confidence intervals. An alternative strategy for countries with limited data on fracture rates is to develop a surrogate model using the hip fracture incidence of a neighboring country and the mortality for the country in question as suggested by the International Society of Clinical Densitometry and International Osteoporosis Foundation [26]. Some comfort may be derived from the logarithmic nature of incidence with age and the similarity of incidence compared with other countries in the region. Thus, a surrogate model would have only a small effect on estimates of fracture probability.

It is relevant, however, that accuracy errors have little impact on the rank order with which the FRAX tool categorizes risk in a given population [27, 28] but they do change the absolute number generated and thus have implications where treatment guidelines are based on cost-effectiveness or the economic burden of disease. In order to address these limitations, populations representative of the general population at risk would need to be studied prospectively, preferably over a 10-year time horizon.

In summary, a FRAX model has been created for the State of Qatar based on a national estimate of the incidence of hip fractures. The model should enhance accuracy of determining fracture probability among the Qatari population and help to guide decisions about treatment.
